# An Effective Extension of Anti-Collision Protocol for RFID in the Industrial Internet of Things (IIoT)

**DOI:** 10.3390/s18124426

**Published:** 2018-12-14

**Authors:** Israel Eduardo de Barros Filho, Ivanovitch Silva, Carlos M. D. Viegas

**Affiliations:** 1 Postgraduate Program in Electrical and Computer Engineering, Federal University of Rio Grande do Norte, Natal 59078-970, Rio Grande do Norte, Brazil; 2 Digital Metropolis Institute, Federal University of Rio Grande do Norte, Natal 59078-970, Rio Grande do Norte, Brazil; ivan@imd.ufrn.br; 3 Department of Computer Engineering and Automation, Federal University of Rio Grande do Norte, Campus Universitário, Natal 59078-900, Brazil; viegas@dca.ufrn.br

**Keywords:** IIoT, DFSA, RFID, throughput

## Abstract

The Industrial Internet of Things (IIoT) is often presented as a concept that is significantly changing industry, yet continuous improvements in the identification and automation of objects are still required. Such improvements are related to communication speed, security, and reliability, critical attributes for industrial environments. In this context, the radio-frequency identification (RFID) systems present some issues related to frame collision when there are several tags transmitting data. The dynamic framed-slotted ALOHA (DFSA) is a widely used algorithm to solve collision problems in RFID systems. DFSA dynamically adjusts the frame length based on estimations of the number of labels that have competed for slots in the previous frame. Thus, the accuracy of the estimator is directly related to the label identification performance. In the literature, there are several estimators proposed to improve labels identification accuracy. However, they are not efficient when considering a large tag population, requiring a considerable amount of computational resources to perform the identification. In this context, this work proposes an estimator, which can efficiently identify a large number of labels without requiring additional computational resources. Through a set of simulations, the results demonstrate that the proposed estimator has a nearly ideal channel usage efficiency of 36.1%, which is the maximum efficiency of the DFSA protocol.

## 1. Introduction

The Industrial Internet of Things (IIoT) is a new industrial concept that combines intelligent and autonomous machines, advanced predictive analytics, and machine–human collaboration to improve productivity, efficiency, and reliability. IIoT provides a world where smart, connected, embedded systems and products operate as part of larger systems [1,2,3,4]. This way, there is expected cost reduction and increased productivity in industry. A foundational technology for IIoT is the radio-frequency identification (RFID), which allows microchips to transmit identification data to a reader via wireless communication [5].

In recent years, RFID technology has been identified as leading a new generation of identification systems due to its reliability, rapid identification, and low cost. RFID aims to boost efficiency in the automation process. Industrial environments have experienced a real advance, due to the use of innovations such as accurate control systems, intelligent sensors, cloud software and information transmission [6]. In this sense, RFID systems minimize errors and improve productivity. All these components are part of the Internet implementation of things in industry.

However, despite RFID systems advantages, when there is a larger number of labels to identify, the greater is the probability of delays and errors in the identification process, compromising the system performance due to collisions. Thus, an efficient protocol is needed to assist in the identification process, especially when there is a huge number of labels.

Dynamic frame slotted ALOHA (DFSA) has been widely used for RFID [7]. In this protocol, the reader organizes the time into one or more frames, and each frame is subdivided into time slots. Labels must be transmitted into only one slot per frame until fully identified by the reader. The length of subsequent frame to the initial frame is dynamically adjusted according to the estimation of the group of labels that competed for slots in the previous frame [8]. This way, both the accuracy of estimating tag population and the adjustment of new frame length may affect the DFSA performance.

In general, the proposed estimators in the literature seek to improve the performance of DFSA. In [9] it is highlighted an efficient estimator compared to its counterparts: Schoute [10], Vogt [11], Eom-Lee [9] and Chen [12]. However, when considering the identification process of 10,000 labels, some estimators require a considerable amount of computational resources (such as CPU and memory). Thus, both complexity and performance should be taken into consideration when implementing the algorithm in an RFID tag, especially those with single-chip microprocessor.

All information is stored on an electronic RFID microchip. The data stored on the tag’s chip will depend on the application. The purpose of a tag is to identify a particular object based on a unique identifier, called an ID or unique item identifier (UII) or even an electronic product code (EPC) code [13]. According to standardized tags, the IDs with the EPC standard provide an improvement in the RFID system, facilitating integration with global networks. To meet the requirements to deploy multiple labels in industry, RFID increasingly requires labels with larger IDs [14]. Once an ID is written to the label’s microchip, it can be read or even changed (if needed). The main precondition for the EPC standard is that all tags have a unique ID in the reader’s interrogation area, since IDs are not always evenly distributed.

The main problem for some tag anti-collision protocols is how the distribution and the size of IDs are performed [15]. This way, tag anti-collision protocols in which IDs are randomly organized suffer from performance loss, increased latency and power consumption, and other issues. These issues occur in tree-based protocols since the tag response is contingent on the requests made by the reader. In an industrial environment that acts heterogeneously, many protocols do not know how to skip unnecessary queries, increasing the number of tags collision. Based on this problem, in this paper we decided to work with ALOHA-based protocols, since the problem of distributing IDs to tags in a heterogeneous manner does not affect the protocol performance.

This paper presents an extension for an estimator for ALOHA-based anti-collision protocols to identify a large number of labels. The proposal presents an ageing factor with an empirical value that acts as a factor rewarding or penalizing the successful slots according to the performance in the previous identification process. When considering industrial environments, there is a relevant problem related to the tags identification in medium access control (MAC) layer, especially when there is a huge number of labels to identify.

To evaluate the performance of the proposed estimator, a discrete event simulator was developed in C/C++ language. This simulator uses as reference the *EPCglobal UHF Class-1 Gen-2* parameters adopted for DFSA passive label anti-collision protocols. The simulator implements an error-free channel, where a tag reader performs queries towards several tags.

The proposal addresses several contributions to solve the problem of tag collisions, being efficient and suitable for multiple scenarios. When compared to other approaches presented in the literature, it is possible to highlight some advantages of our estimator:

1. It can identify a large number of tags, presenting a low computational cost and good performance;
2. It presents an improved throughput, since it can reduce the number of colliding slots;
3. It uses an ageing factor that acts as a factor rewarding or penalizing the successful slots according to the performance in the previous identification process;
4. It is standard-compliant to *EPCglobal UHF Class-1 Gen-2*.

The remainder of the paper is organized as follows: Section 2 presents the basics of RFID systems, by discussing its functionalities, type of tags, collisions occurrence, MAC and common applications. Section 3 presents the ALOHA-based anti-collision protocol (DFSA) and reviews some other estimators proposed in the literature. Section 4 describes our proposed DFSA estimator, which is an extension to Chen’s estimator. Section 5 evaluates the performance of our estimator in different scenarios. Finally, Section 6 concludes the paper and presents avenues for future research.

## 2. Radio-Frequency Identification (RFID)

Currently, there is a demand for manage multiple objects with higher speed and increased accuracy. Based on this demand, RFID technology appears to be appropriate to perform tasks such as identification, tracking, and automation of objects. An advantage of RFID over other identification systems is related to reading range, since it is not necessary the approximation of the object for its proper identification [7].

**Figure 1:**
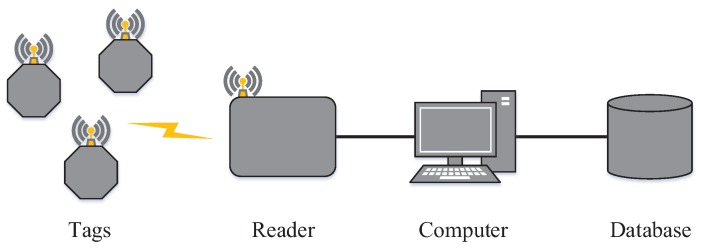
Basic RFID system.

A basic RFID system consists of three main elements: readers, labels (or tags) and a database [16]. Figure 1 presents a basic RFID system. The reader is a device whose function is to identify the labels and, consequently, extract information from them. It uses radio-frequency waves for communication. Moreover, the reader controls the labels medium access, which is carried out by anti-collision protocols.

The database is the central entity of an RFID system. It has the role of processing and storing information according to the needs of each application. In general, it is a database in which information is stored and accessed by labels and readers.

Labels are the simplest elements of the system. They store information of objects which are associated with them. They have a unique identifier by which they can be identified. It is worth noting that passive tags are increasingly being used in RFID systems in industry. They are small, simple to manufacture, consequently, cheap. Moreover, passive tags are durable, since they do not have internal battery. Due to these characteristics, this paper is focused on passive tags only.

### 2.1. Collisions in RFID Systems

Since RFID systems use wireless medium for communication, there are some problems that arise from shared medium, such as collisions and signals interference [17,18,19,20]. In general, collisions may compromise the RFID systems communication, since errors in label identification may occur. Moreover, when collisions occur, a retransmission strategy is employed, which increase energy and bandwidth consumption [21]. When considering passive tags, the collision problem can be exacerbated by its computational limitation. In most cases, the communication becomes impossible. This is a problem that might be address.

There are two types of collisions that need to be resolved: collisions among readers and collisions among tags. Collisions among readers occur when the signals of two or more readers overlap [22], making it confusing for the tags to identify to which reader they should respond to. On the other hand, collisions among tags occur when two or more labels send information simultaneously to a single reader.

There are defined four MAC protocols: space division multiple access (SDMA), code division multiple access (CDMA), frequency division multiple access (FDMA) and time division multiple access (TDMA).

The SDMA protocol [23] has the function of distributing different frequency bands to different neighboring regions. This method is widely used to perform signal coverage among adjacent cells, such as in the cellular telephone network. However, its use in RFID systems is unfeasible due to the use of several sectoral antennas and readers, thus increasing the complexity of the system [7].

In the CDMA protocol [6], uniformly distributed spectral propagation techniques are applied to the network elements with the same power and frequency. Each element has a different code to modulate the signal. However, when considering its use in RFID systems, this protocol presents some problems, since code division requires a high computational cost.

The FDMA protocol divides the available bandwidth into multiple frequency bands. Each user has a reserved frequency band, which can be used until the end of its transmission. However, the use of several frequency bands increases the tags and readers cost [24].

In the TDMA protocol [25], the channel is divided into several fixed-size time slots, where each element transmits in a given slot at a time, thus avoiding interference. Due to its simplicity and low computational cost, this protocol has become the most suitable option for RFID systems. By using TDMA, the available time intervals for labels transmission is classified in three ways: empty slots, when there are no transmissions; successful slots, when only one tag can transmit its identification; and colliding slots, when two or more tags attempt to transmit simultaneously within the same time slot.

To solve the issues caused by tags collision, several protocols have been identified in the literature [22]. Given passive tags computational limitation and their potential use in the industrial market, its low cost renders it suitable to use TDMA as MAC in RFID systems. Moreover, TDMA can be divided into two categories of anti-collision protocols: tree-based protocols and ALOHA-based protocols. Being the latter, the most common for IIoT [5].

### 2.2. Overview of DFSA in EPCglobal UHF Class-1 Gen-2

The DFSA anti-collision protocol was adopted by the *EPCglobal UHF Class-1 Gen-2* standard to solve passive tags collision problem for RFID systems [26]. Its main strategy is to enable a dynamic adjustment of frame length for each reading cycle in label identification [27,28,29]. This adjustment impacts on the DFSA performance, that is directly related to the frame length. As stated, the reader has the function of coordinating medium access, and thus it dynamically adjusts the frame length at each identification cycle. However, this also depends on the estimation of labels population that compete for time frames.

In the DFSA implementation, in addition to MAC, there are some commands to identify a group of labels: *Select*, to select a group of tags to be identified; *Query*, to identify the tag or a group of them; *QueryAdjust*, to adjust the frame length; *QueryRep*, to repeat the query; and *Ack*, to acknowledge frame transmission.

First, DFSA works with the reader triggering a transmission to identify a group of labels within its coverage radius, by sending *Select* command. Then, it sends a *Query* command to identify the selected tags, which specifies the minimum and maximum number of tags related to the initial frame length. The frame must be a power of 2. When a tag receives a *Query* command, it generates a 16-bit random number (RN16), and extracts part of a subset of *Q*-bit queries, generating a range interval counter. This counter is decremented by 1 as tags receive a *QueryRep* command. When this counter reaches zero, the tag sends its RN16. Actually, the real ID of a tag is 96-bit EPC. Thus, the RN16 can be considered as a temporary ID to reduce the collision interval. Having sent the *Query* command, the reader starts to check each record of each time interval for a possible RN16 communication reception.

**Figure 2:**
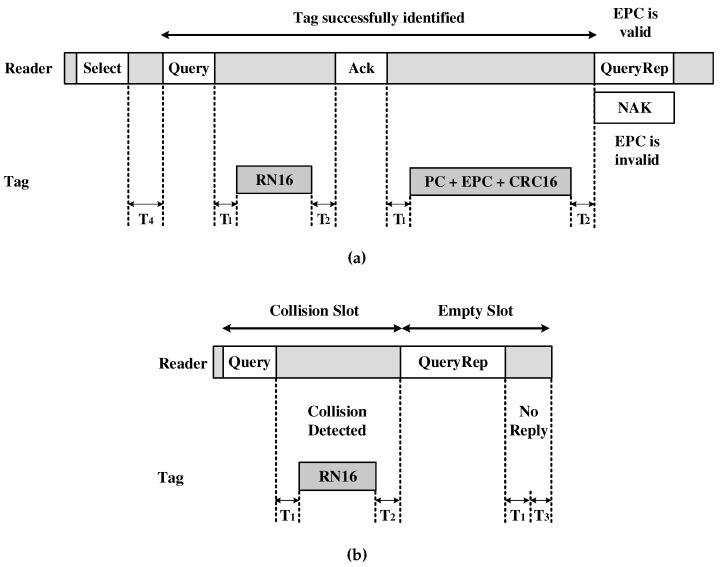
Different tags response. (**a**) Tag response successfully identified, and (**b**) Response of a colliding slot and an empty slot.

For a given time slot, there are possibilities: successful slot, when the tag is successfully identified; slot in collision, when two or more tags competed for the same time interval; and empty slot, when there is no transmission (see Figure 2a,b). When a slot or tag is successfully identified, the reader acknowledges the tag with an ACK. Then, the tag transmits its 96-bit EPC to the reader. If there is a collision of RN16, hence no tag identified, the reader finishes the transmission time interval, checks all time slots of frame $2^{Q}$, and starts a new round of *Query* transmissions with a new *Q* updated.

### 2.3. Application Scenarios

RFID system applications include supply chain management, retail, aircraft maintenance, document fraud, baggage handling and healthcare [30,31,32,33]. Several organizations are exploring RFID in their core operations to leverage automation processes. For instance, Walmart reduced inventory shortfalls by an average of 30% following the launch of its RFID program [34]; Throttleman solved problems with space constraints in storage [35]; Metro Group achieved improved control in handling materials [34]; and Cold Chain Logistics has been able to significantly reduce the damage caused by cold container failures due to poor internal temperature monitoring [36]. The main results are displayed in Table 1.

## 3. Related Works

**Figure 3:**
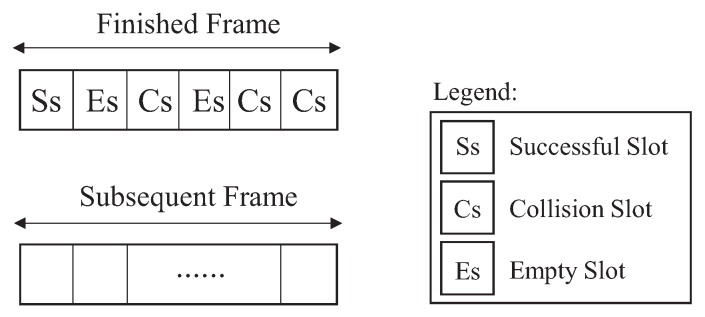
Frames using DFSA.

In the literature, there are several studies for DFSA estimators, including Chen [12], Eom-Lee [9], Vogt [11] and Schoute [10], which will be discussed below. For this, we consider an operation example of DFSA algorithm, depicted in Figure 3. There are two frames of interest, the finished frame, and the subsequent frame. The finished frame represents the number of labels that competed for slots in that frame, where Ss, Es and Cs correspond to the number of successful slots, empty slots, and colliding slots, respectively.

The length of the previous frame is based on the tag population that competed in the finished frame, which will be invoked if there is at least one slot in collision to trigger the estimator and thus generate the next frame. In this case, the size of the previous frame will be represented by $\hat{f}$. For all estimators described in this paper, $\hat{n}$ represents the number of estimated labels that competed for slots in the finished frame.

### 3.1. Schoute

The Schoute estimator [14] calculates the length of previous frame by multiplying the number of colliding slots in the frame with a factor of 2.39. This factor is the approximate value of the number of tags that will be transmitted in each collision slot in the finished frame. The length of the previous frame ($\hat{f}$) is given by:

(1) $$\begin{array}{c} \hat{f}=2.39\times Cs \end{array}$$

Equation (1) is obtained from random labels arrival, modelled by a Poisson distribution. This is an approximate probability and it is not optimal, since variations in estimated values in relation to real values may occur. To estimate the labels that contended for slots, $\hat{n}$ will be equal to the sum of successfully slots (Ss) with the length of the previous frame ($\hat{f}$) as follows:

(2) $$\begin{array}{c} \hat{n}=Ss+2.39\times Cs \end{array}$$

### 3.2. Vogt

To estimate the number of tags that competed in the finished frame, the concept of probability is used, where allocation in slots is carried out randomly in the current frame [15]. This way, the expected quantity of *r* tag transmissions in a frame length *L* is binomially distributed, where *n* represents the number of tags that competed for slots in the frame, represented by the following equation:

(3) arL,n=Lnr1Lr1−1Ln−r

The Vogt method is based on the Chebyshev inequality, where the result of an experiment involving a random variable *x* is close to the expected value of *x* [11]. Thus, Vogt defines the function $\hat{n}$ to estimate the number of labels and seeks to minimize the distance between the array <Es;Ss;Cs> and the array containing the expected values for Es;Ss;Cs. This function is given by:

(4) $$\begin{array}{c} \hat{n}\left(L,Es,Ss,Cs\right)={}_{\;n}^{min}\left|\left(\begin{array}{c} a_{0}^{L,n}\\ a_{1}^{L,n}\\ a_{2}^{L,n} \end{array}\right)-\left(\begin{array}{c} Es\\ Ss\\ Cs \end{array}\right)\right| \end{array}$$

Thus, $\hat{n}$ is the value of *n* that minimizes the modulus of the difference of the two arrays representing real values and expected values in the current frame, according to Equation (4). To find the frame length of next frame, Vogt defines a function based on the estimated number of tags, as presented in Equation (5). Possible lengths are given in Table 2. For instance, if $\hat{n}\in \left[51,56\right]$ both $\hat{f}$ = 64 and $\hat{f}$ = 128 are suitable choices.

(5) $$\begin{array}{c} \hat{f}=\hat{n}-Ss \end{array}$$

### 3.3. Eom-Lee

The estimator uses an interactive algorithm to estimate the number of tags competing for slots in a frame, and the length of the subsequent frame [9]. Thus, *L* is the size of the analyzed frame to estimate the size of the next frame. By assuming that *L* is equal to the estimated number of tags that competed multiplied by a factor β to be determined, *L* can be represented by:

(6) $$\begin{array}{c} L=\beta \times \hat{n} \end{array}$$

According to the number of tags competing for collision slots, it is also assumed to be equal to γ. Thus, the length of the next frame is equal to the backlog, so the value of $\hat{f}$ can be calculated as:

(7) $$\begin{array}{c} \hat{f}=\hat{n}-Ss=\gamma \times Cs \end{array}$$

To estimate the number of labels, it is needed to determine β and γ values. In [7], the relationship between both can be explained by Equation (8). The probability of *r* labels to transmit in the same slot can be approximated by a binomial distribution, and the equation can be modelled by a Poisson distribution with mean n/L.

(8) $$\begin{array}{c} \gamma =\frac{1-e^{-\frac{1}{\beta }}}{\beta \left(1-\left(1+\frac{1}{\beta }\right)e^{-\frac{1}{\beta }}\right)} \end{array}$$

Eom-Lee estimator uses an interactive algorithm to estimate β and γ values. Consider that $\beta_{k}$ and $\gamma_{k}$ are, respectively, approximations for β and γ values in the *k*th algorithm iteration. These approximations are obtained according to the following equations:

(9) $$\begin{array}{c} \beta_{k}=\frac{L}{\gamma_{k-1}\times Cs+Ss} \end{array}$$

(10) $$\begin{array}{c} \gamma_{k}=\frac{1-e^{-\frac{1}{\beta_{k}}}}{\beta \left(1-\left(1+\frac{1}{\beta_{k}}\right)e^{-\frac{1}{\beta_{k}}}\right)} \end{array}$$

In the initial step, it is assumed that $\beta_{1}=\infty$ and $\gamma_{1}=2$, and that at each subsequent step *k* a new approximation is determined for β and γ, respectively. Hence, when $|\gamma_{k^{*}-1}-\gamma_{k^{*}}|$ is smaller than a threshold $\in_{threshold}$, the interactive process is interrupted. Thus, the values $\gamma_{k^{*}-1}$ and $\gamma_{k^{*}}$ represent, respectively, the previous and current approximation for γ. Therefore, the length $\hat{f}$ of the next frame and the estimated number of $\hat{n}$ labels are obtained by Equations (11) and (12), respectively, where $\beta_{k^{*}}$ is the current approximation for β value.

(11) $$\begin{array}{c} \hat{f}=\gamma_{k^{*}}\times Cs \end{array}$$

(12) $$\begin{array}{c} \hat{n}=\frac{\hat{f}}{\beta_{k^{*}}} \end{array}$$

### 3.4. Chen

Chen estimator [12] initiates a reading round with a *Q* parameter, where the number of slots Es, Ss, Cs are counted regarding the tags that competed in the initial frame. The estimated number of tags $\hat{n}$ is still applied in the same initial frame, represented by:

(13) $$\begin{array}{c} \hat{n}=\left(Ss+k\times Cs\right)\times \frac{L}{i} \end{array}$$

where *k* is 2.39, which corresponds to an expected number of colliding slots. Moreover, with the result of $\hat{n}$ and the frame length (*L*), the reader can check if the estimated number of labels is within the optimal interval, as listed in Table 3.

If $\hat{n}$ does not match the optimal interval, the frame length will be adjusted to find the appropriate size based on the backlog, calculated as follows:

(14) $$\begin{array}{c} backlog=\hat{n}-Ss \end{array}$$

In fact, the frame length may impact the efficiency of the estimator used. Based on the relationship between frame length and average number of labels, it is possible to calculate the average efficiency for each frame length, so that it does not compromise the performance of the estimator. These values can be found in Table 3, by using Equation (15).

(15) $$\begin{array}{c} {\bar{U}}_{Q}=\frac{1}{\left(n_{Q_{2}}-n_{Q_{1}+1}\right)}\sum_{n=n_{Q_{1}}}^{n_{Q_{2}}}\frac{n}{2^{Q}}\times {\left(1-\frac{1}{2^{Q}}\right)}^{n-1} \end{array}$$

## 4. Proposed Extension for Estimator

The *EPCglobal UHF Class-1 Gen-2* standard adopts DFSA algorithm to address tags collision problem. However, it does not specify how to dynamically adjust the frame length [35]. In this sense, this section presents a comprehensive and easy-to-implement approach to perform a frame length adjustment in DFSA protocol.

We consider that *n* labels need to be identified, and their frame length is *L*. We assume that the number of labels allocated in a given time slot can be modelled as a binomial distribution, where $\bar{U}$ represents the channel usage efficiency (also called normalized throughput):

(16) $$\begin{array}{c} \bar{U}=\frac{n}{L}\times {\left(1-\frac{1}{L}\right)}^{n-1} \end{array}$$

Table 4 establishes a relationship between the frame length and tag population. For instance, when the amount of tags is in the interval of 90 to 177 (when Q=7), the frame length must be 128 slots to increase system throughput. For a given number of labels, the appropriate frame length can be obtained from Equation (16).

This constrained frame length will cause a reduction in channel efficiency when compared with the ideal case, in which the frame length is set according to the number of labels. Under this constraint, the average channel efficiency is given by $n_{Q_{1}}$ and $n_{Q_{2}}$ when they are the lower and upper limits, respectively, for a given frame length $2^{Q}$. Based on the relationship between the frame length and tag population listed in Table 4, the average channel efficiency can be obtained for each frame size used Equation (15).

For instance, we consider that the number of labels is evenly distributed between 1 and 10,000. The maximum channel efficiency is 36.1%. This result means that the size of the constrained frame can lead to reductions of only 4.3% in performance. Since the frame size is a power of 2, the average channel efficiency is 36.1%. It can be considered as the optimal maximum performance of *EPCglobal UHF Class-1 Gen-2* for collision avoidance.

This strategy provides a small performance improvement; however, it requires more computational resources. Therefore, we use the reader as the medium access coordinator, to adjust the subsequent frame length during the reading process, and to detect improper frame lengths before the end of each reading cycle. The latter can be treated as a rapid adaptation of the frame length. A simple, but efficient method is to reduce the computational complexity to be able to estimate the number of tags in collision. Based on the Schoute method, we propose an extension to estimate the number of tags in such a way that the tag accumulation is estimated according to the number of collision slots.

The proposed anti-collision protocol is shown in Figure 5. At first, the reader begins a reading cycle of tags, broadcasting a query command *Q* for all tags within the its coverage area. This command corresponds to frame length $2^{Q}$, meaning that there are $2^{Q}$ intervals in the current reading frame. Thus, in the last time slot, the reader starts recording the number of empty slots Es, the number of slots successfully identified Ss, and the number of slots in collision Cs. An estimated number of labels is then generated to be read at the beginning of the reading cycle according to Equation (17):

(17) $$\begin{array}{c} \hat{n}=\frac{L}{i}\times \left(\alpha \times Ss_{i}+k\times Cs_{i}\right) \end{array}$$

where *k* represents a coefficient, *L* the frame length, and *i* the *i*th time slot. Considering that there are at least two tags involved in a collision, the coefficient *k* can be adjusted to be 2 as a minimum limit measure to perform tag estimation. To minimize complex implementations, we considered k=2.39, which is the same coefficient used in the Schoute method.

Moreover, the proposal presents an ageing factor (α) that acts rewarding or penalizing Equation (17), according to the performance of the previous schedule. The factor α, is multiplied by the number of successful slots. This factor is the average representation of the number of successful slots in the previous frame, which means that the number of tags that had successful identification will directly impacts on the current frame. The average is the ratio between the number of successful slots over the total number of slots in a frame.

Initially, the ageing factor α equals 1. There is no past, since the identification process is starting. In the following rounds the value of α can be changed assuming values between the interval 0.8 and 1.2. These values were empirically chosen, among others, since they presented a better performance for the ageing factor scheme. When α is 0.8, it indicates that the number of successful slots in the previous frame is below the expected average, and then it will be penalized. On the other hand, if the next frame has a value greater than or equal to the average of successful slots, α will be equal to 1.2, which means a reward for achieving several successful slots in the previous round. This technique makes it easy to estimate a successful slot according to the past schedule.

**Figure 4:**
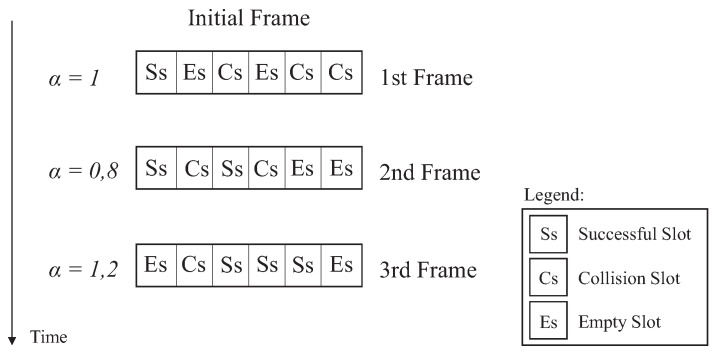
Ageing Factor.

Figure 4 exemplifies the ageing factor process, where the initial frame starts with α=1. Since in the past there was any frame, so the second frame will receive penalizing factor of α=0.8. By calculating the average successful slots in the second frame, the third frame will be rewarded with α=1.2.

**Figure 5:**
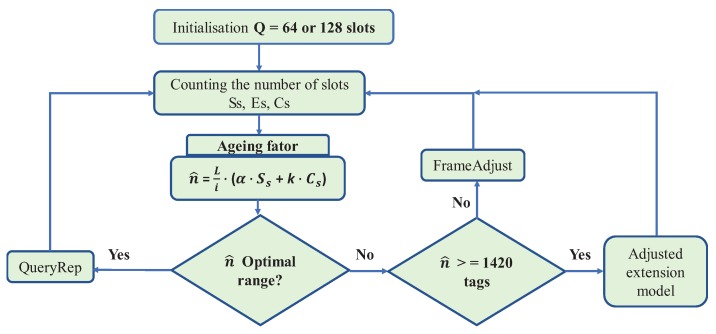
Flowchart of the proposed algorithm.

The reader should check whether the tags estimation is within the interval associated with frame length *L*, as listed in Table 4. If not, and if the tag number is ≥ 1420 the reader will adjust the frame length by sending a *FrameAdjust* command to increase or decrease the number of slots in the next frame. Otherwise, if $\hat{n}$ is less than 1420 labels then an extension template will be applied according to Table 4, by sending a *QueryRep* command. Figure 5 illustrates the proposed algorithm.

If a frame length adjustment is required, the reader needs to set a suitable length, based on the estimated backlog, and to estimate the number of tags according to the number of tags successfully identified (from Equation (14)). Based on the backlog estimation, the reader adjusts the frame length using *QueryAdjust* command or a *Q* value based on Table 4. However, if the frame length adjustment does not occur during the identification process, the reader adopts the Schoute estimator 2.39×Cs to estimate the delay, where Cs is the number of collision slots at the end of the current frame.

According to the estimators presented in Section 3, it was observed that the Chen estimator is the simplest. To increase the estimator precision, it is necessary a significant computational load to perform the mathematical calculations. Although a more accurate estimator improves the DFSA algorithm performance, it may lead to high costs, especially when the number of labels is huge, such as a population of 10,000 tags, for instance.

**Figure 6:**
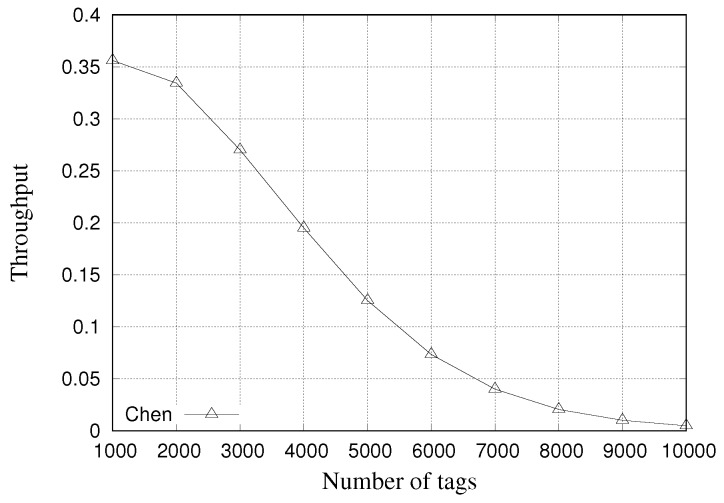
Throughput when using Chen estimator with different tag population.

However, Chen estimator is only able to efficiently estimate if the maximum number of labels is up to 1420 tags. Above that, the estimator loses performance since it estimates a wrong length for next frame to accommodate such tag population. Figure 6 illustrates Chen estimator performance, where it can be seen the throughput worsen as the number of tags increase. Thus, Chen estimator is not adequate to estimate a huge number of tags.

Seeking to improve Chen estimator performance, it is proposed an extension able to identify a tag population above 10,000, without compromising the computational performance. It is important to note that to implement the extension, some values have been added to the estimator through the proposed Equation (18) and the size of the next frame remains $2^{Q}$.

(18) $$\begin{array}{c} \begin{array}{c} n_{Q_{1}}={\overset{´}{n}}_{Q_{2}}+1\\ n_{Q_{2}}=\left({\overset{´}{n}}_{Q_{1}}\times 2\right)-2 \end{array} \end{array}$$

where $n_{Q_{1}}$ is the start of the new label interval, and $n_{Q_{2}}$ is the end of the new label interval. Thus, ${\overset{´}{n}}_{Q_{1}}$ and ${\overset{´}{n}}_{Q_{2}}$ correspond to the beginning and the end of the previous interval, respectively. Therefore, Equation (18) demonstrates how better perform the proposed estimator. Its performance can be proved by Equation (15).

This way, the estimator can identify a tag population above 10,000, maintaining a channel efficiency of 36.1%. Moreover, it does not require additional computational resources nor complex mathematical calculations, being a simple, fast, and efficient estimator. It can be implemented in a low-level hardware label and be used in conjunction with the DFSA anti-collision protocol without issue. Thus, the proposal is suitable for an industrial environment, where a large number of RFID tags is required.

## 5. Performance Evaluation

This section presents a performance evaluation of the proposed estimator, and it is compared with other estimators available in the literature (see Section 3). To analyze the estimator performance, a discrete event simulator was developed in C/C++ language. This simulator models an RFID system scenario, where there are several tags and one reader, sharing an error-free channel. To ensure convergence, we performed 4000 simulations rounds.

**Figure 7:**
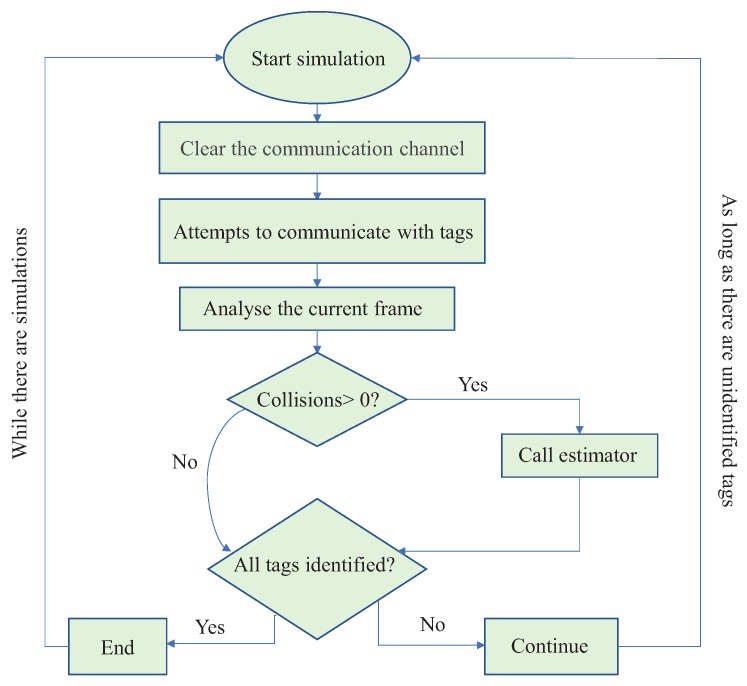
Simulator flowchart.

Figure 7 illustrates a flowchart of how simulator was modelled. A simulation is initiated by clearing the communication channel, which is implemented as an array and each element is equivalent to a time slot. Then, the RFID tag reader sends a signal to each tag, and then waits for responses. We considered each array element as a counter, where its value is incremented by 1 when there is a tag response. Each tag is defined to respond only once. In the end, each slot in the array will contain the number of response attempts. Obviously, if there is a slot with 0 responses, it is considered an empty slot. A slot whose value equals 1 is considered a successful slot, and a slot whose value is greater than 1 is considered a colliding slot.

Once the communication is started, current frame is analyzed to verify the collisions occurrence. If there is no collision, there is no need to adjust frame length. However, if there is a collision, a call to the estimator is made and a new frame length is computed.

The identification time varies since there are different slot times duration, depending on whether they are successful, collision or empty. Table 5 presents approximate values of each slot time. These values are based on the *EPCglobal UHF Class-1 Gen-2 standard* for passive UHF systems.

To obtain the required time to communicate with all tags, we need to compute all time slots and commands used in the communication process. The parameters used in the simulation process for this computation are shown in Table 6. These parameters are defined according to the *EPCglobal UHF Class-1 Gen-2 standard*. It is worth noting that in our simulator the time to select tags ($T_{4}$) is not considered, since we consider all tags in the simulation scenario as selected.

The metrics considered for this performance evaluation comprises: the number of empty slots, the number of slots in collision, the number of slots needed to identify all labels, and channel throughput. Moreover, most evaluations presented in the literature considers only a tag population of up to 1000. In this performance evaluation we considered a tag population of up to 10,000 tags.

We chose to use initial frames with 64 and 128 slots since they present better behavior in relation to the metrics mentioned in the paper, when considering a scenario with large number of labels. These values are also widely adopted in literature.

### 5.1. Simulation Results for Slots in Collision

**Figure 8:**
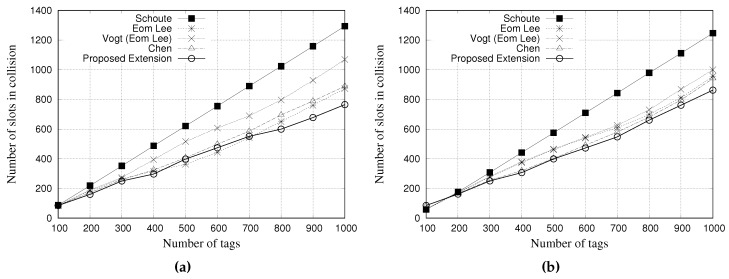
Simulation results for slots in collision when using different estimators with a tag population of 1000 tags. (**a**) Frame length of 64 slots, and (**b**) Frame length of 128 slots.

**Figure 9:**
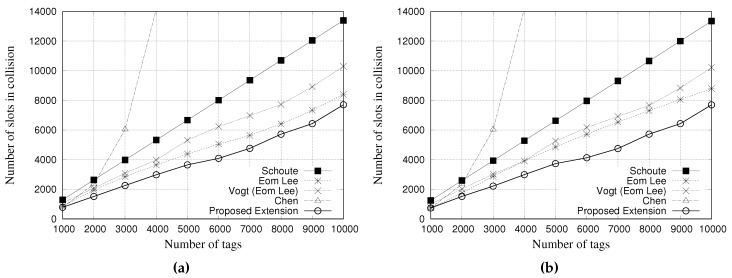
Simulation results for slots in collision when using different estimators with a tag population of 10,000 tags. (**a**) Frame length of 64 slots, and (**b**) Frame length of 128 slots.

A collision slot occurs when two or more tags contend simultaneously for the same slot. Figure 8 and Figure 9 present the performance evaluation of multiple estimators regarding the number of slots in collision. Figure 8a,b show the results for a tag population of 1000 tags, with frame lengths of 64 and 128 slots, respectively. Figure 9a,b show the results for a tag population of 10,000 tags, with frame lengths of 64 and 128 slots, respectively.

The results show that, regardless of the frame length of 64 or 128 slots, Schoute estimator presents a larger number of slots in collision when compared with other estimators. On the other hand, our proposed estimator can reduce the number of slots in collision. These results indicate that our proposed ageing factor can adjust the subsequent frame accordingly, to avoid collisions.

From the results of Figure 9, regardless of the frame length, Chen estimator presents the worst performance, since it is not able to correctly estimate tag population above 3000. Consequently, the frame length is not adjusted to accommodate such number of labels. As it can be seen, other estimators were able to adjust the frame length to accommodate a tag population up to 10,000. However, there are some performance differences among estimators. Schoute estimator presented a slightly worst performance when compared with Eom-Lee, Vogt, and our proposed estimator. On the other hand, our proposed estimator presented the best performance. Regardless of the frame length of 64 or 128 slots, the proposed estimator is less prone to collisions, even considering a large tag population. The ageing factor seems to be more accurate in estimating subsequent frame length based on previous frame, thus leading to less collisions.

### 5.2. Simulation Results for Empty Slots

**Figure 10:**
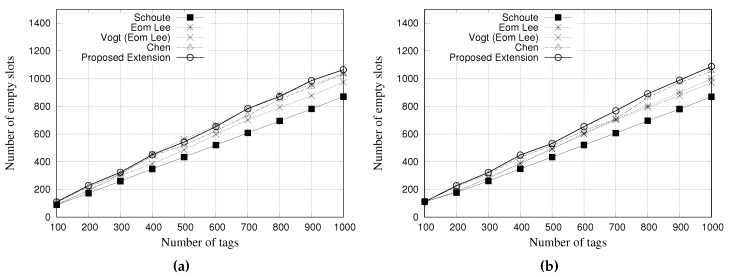
Simulation results for empty slots when using different estimators with a tag population of 1000 tags. (**a**) Frame length of 64 slots, and (**b**) Frame length of 128 slots.

**Figure 11:**
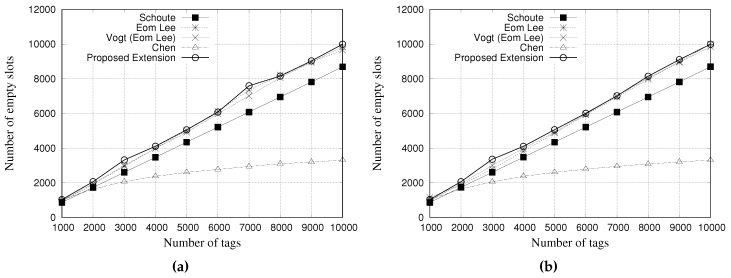
Simulation results for empty slots when using different estimators with a tag population of 10,000 tags. (**a**) Frame length of 64 slots, and (**b**) Frame length of 128 slots.

An empty slot occurs when none tag contended for that slot. Figure 10 and Figure 11 present the performance evaluation of multiple estimators regarding the number of empty slots. Figure 10a,b show the results for a tag population of 1000 tags, with frame lengths of 64 and 128 slots, respectively. Figure 11a,b show the results for a tag population of 10,000 tags, with frame lengths of 64 and 128 slots, respectively.

The results indicate that the Schoute estimator can reduce the number of empty slots in both scenarios. It is justified by the multiplication of the number of colliding slots by the constant of 2.39 to generate the subsequent frame. This reduces the number of empty slots but increases the number of slots in collision.

Despite Chen estimator as several slots in collision, here it presents fewer empty slots when compared with the other estimators. It is important to note that Chen estimator has a limitation or maximum size of its subsequent frame, which consequently impacts on this result.

Again, the results of other estimators are very similar, but our proposal presents a small reduction in the total number of slots when compared with other estimators.

### 5.3. Simulation Results for Total Number of Slots

**Figure 12:**
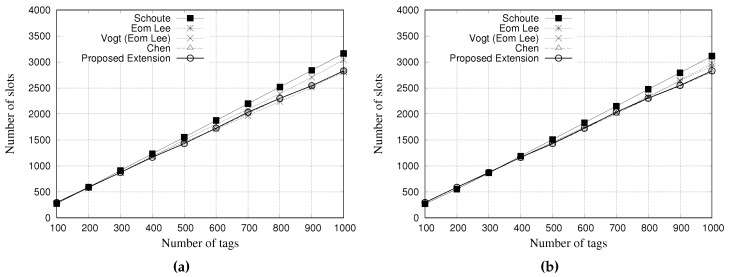
Simulation results for total number of slots when using different estimators with a tag population of 1000 tags. (**a**) Frame length of 64 slots, and (**b**) Frame length of 128 slots.

**Figure 13:**
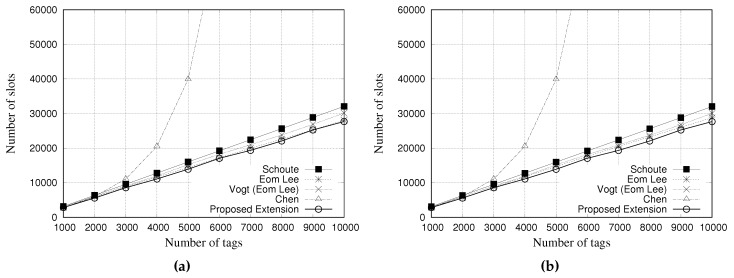
Simulation results for total number of slots when using different estimators with a tag population of 10,000 tags. (**a**) Frame length of 64 slots, and (**b**) Frame length of 128 slots.

This performance evaluation considered the total amount of slots used by each estimator during the simulation process. Figure 12 and Figure 13 present the performance evaluation of multiple estimators regarding the total number of slots. Figure 12a,b show the results for a tag population of 1000 tags, with frame lengths of 64 and 128 slots, respectively. Figure 13a,b show the results for a tag population of 10,000 tags, with frame lengths of 64 and 128 slots, respectively.

Regarding the results with 1000 tags, all the estimators presented the same performance in relation to the total number of slots used, regardless of the frame length.

The results presented in Figure 13a,b, indicate that Chen estimator needs to use more slots to identify all tags. Above 5000 tags, the number of slots is greater than 60,000, presenting a degradation in communication performance. This result, and the above presented, indicate that Chen estimator is not suitable for large a tag population.

Our proposed estimator presented a performance very similar to the other estimators, by using almost the same quantity of slots to read all tags. In this scenario, there are almost no difference among estimators, except Chen’s one.

### 5.4. Simulation Results for Throughput

**Figure 14:**
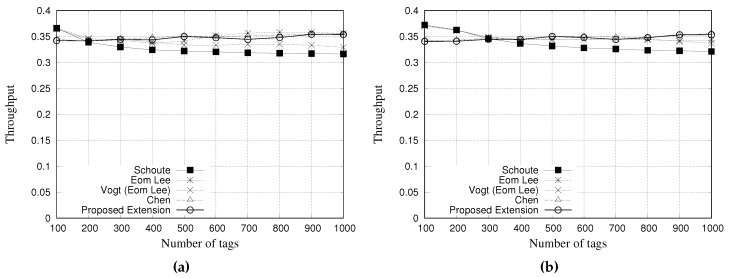
Simulation results for throughput when using different estimators with a tag population of 1000 tags. (**a**) Frame length of 64 slots, and (**b**) Frame length of 128 slots.

**Figure 15:**
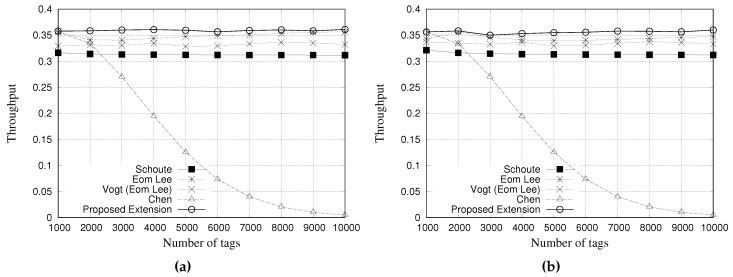
Simulation results for throughput when using different estimators with a tag population of 10,000 tags. (**a**) Frame length of 64 slots, and (**b**) Frame length of 128 slots.

The throughput is calculated as Equation (19):

(19) $$\begin{array}{c} T_{hput}=\frac{N_{Ss}}{N_{Ss}+N_{Cs}+N_{Es}} \end{array}$$

where $N_{Ss}$, $N_{Cs}$ and $N_{Es}$ are the average number of successful slots, colliding slots, and empty slots, respectively. Figure 14 and Figure 15 present the simulation results of multiple estimators regarding the throughput. Figure 14a,b show the results for a tag population of 1000 tags, with frame lengths of 64 and 128 slots, respectively. Figure 15a,b show the results for a tag population of 10,000 tags, with frame lengths of 64 and 128 slots, respectively.

From the results with 1000 tags, it is possible to note that all the estimators presented a similar throughput. Vogt and Eom-Lee presented a slightly lower throughput when compared with Chen and our proposed estimator, despite the results being very similar.

From the results with 10,000 tags, as it can be seen, the Chen estimator presents a low throughput performance when compared with other estimators. This behavior is justified by the large number of slots needed to identify all tags. Regarding our proposed estimator, it presents an improved performance when compared to others, despite being very similar.

## 6. Conclusions

Today, RFID technology has become a reliable solution for tracking objects in a diverse kind of scenarios, including critical industrial environments. However, there are open issues related to the performance of RFID systems when considering large number of tags. The typical anti-collision solutions based on framed-slotted ALOHA are inefficient to reach acceptable results considering a larger scale of tags such as 10,000. The problem emerges because the anti-collision estimators require complex mathematical calculations to obtain a more accurate solution, and this is prohibitive due to hardware limitations of passive tags. Solutions to decrease the computational complexity of anti-collision estimators had been investigated in the literature. However, they are unable to efficiently identify several tags greater than 1240.

In this paper, we proposed an extension for an anti-collision estimator which can communicate with a tag population of up to 10,000 tags. A simulation module was developed to evaluate the performance of estimators in different scenarios. The results demonstrated that our proposed extension presents an improved performance when compared with other estimators, regardless of the tag population of up to 1000 or 10,000 tags. The reduction of collisions impacted directly in the throughput improvement. The ageing factor mechanism acted as a filter that smooth the collisions, even though when the transmission rate increases in the communication channel.

In future works, we intend to support a dynamic ageing factor to improve the performance of anti-collision estimator. The idea is to learn the channel behavior according to a sliding window with a limited size.

## Figures and Tables

**Table 1:** RFID technology application scenarios.

Segment	Application	Estimated Number of Tags
Retail [34]	• Reduction of almost 90% for pallet mounting; • Increase in supply; • Decrease in manual processes as well as errors record; • Increased productivity and customer satisfaction; • Errors reduction with the extinction of barcode; • Improved efficiency in product monitoring; • Current activities status; • Availability of product information.	Hundreds
Apparel [35]	• Product theft reduction; • Reduction in the time spent in stock; • Optimal reading rate with an accuracy efficiency of 99.9%; • Fixed storage space problems.	Thousands
Logistics [36]	• Increase in food exports; • Real-time temperature monitoring in containers; • Better product tracking to the end customer; • Damaged products reduction.	Dozens
Industry 4.0 [37]	• Improved identification process in the items receipt; • Improvements in locating tool parts and equipment; • Benefits in items maturity in the manufacturing process; • Safety in the process of loading and unloading products.	Thousands

**Table 2:** Vogt frame length.

$\hat{f}$	$\hat{n}\in \left[x,y\right]$
16	[1, 9]
32	[10, 27]
64	[17, 56]
128	[51, 129]
256	[112, ∞]

**Table 3:** Frame length in relation to the number of tags.

Q	Frame Size $L=2^{Q}$	Tags $n_{Q_{1}}-n_{Q_{2}}$	Channel Efficiency
2	4	[1,5]	0.404
3	8	[6,11]	0.382
4	16	[12,22]	0.372
5	32	[23,44]	0.366
6	64	[45,89]	0.363
7	128	[90,177]	0.362
8	256	[178,355]	0.361
9	512	[356,710]	0.361
10	1024	[711,1420]	0.361

**Table 4:** Frame length in relation to the number of tags.

Q	Frame Length $L=2^{Q}$	Tags $n_{Q_{1}}-n_{Q_{2}}$	Channel Efficiency
2	4	[1,5]	0.404
3	8	[6,11]	0.382
4	16	[12,22]	0.372
5	32	[23,44]	0.366
6	64	[45,89]	0.363
7	128	[90,177]	0.362
8	256	[178,355]	0.361
9	512	[356,710]	0.361
10	1024	[711,1420]	0.361
11	2048	[1421,2840]	0.361
12	4096	[2841,5680]	0.361
13	8192	[5681,11,360]	0.361

**Table 5:** Identification time for different time slots types.

Slot Type	Equation	Value
Successful	$2\times \left(T_{1}+T_{2}\right)+T_{RN16}+T_{EPC+PC+CRC16}$	1375 μs
Collision	$\left(T_{1}+T_{2}\right)+T_{RN16}$	337.5 μs
Empty	$\left(T_{1}+T_{3}\right)$	67.5 μs

**Table 6:** Simulation parameters to compute the reading time of all tags, based on *EPCglobal UHF Class-1 Gen-2 standard*.

Parameter	Description	Value
$T_{rate}$	Transmission rate	80 Kbps
$RT_{Cal}$	Reader/tags calibration time	31.25μs
LF	Subcarrier signal frequency	160 KHz
$T_{pri}$	Pulse repetition interval	$\frac{1}{LF}$
$T_{1}$	Time between reader request and tag immediate response	62.5μs
$T_{2}$	Tag response time	62.5μs
$T_{3}$	Waiting time after $T_{1}$ before transmitting another command	5μs
$T_{4}$	Interval to select tags	112.5μs
$T_{q}$	Query time	421.5μs
$T_{RN16}$	RN16 time	212.5μs
$T_{ACK}$	ACK time	337.5μs
$T_{EPC+PC+CRC16}$	EPC/PC/CRC16 time	912.5μs

## Abbreviations

The following abbreviations are used in this manuscript:

CDMA	Code division multiple access
CRC16	16-bit cyclic redundancy check
Cs	Collision slot
DFSA	Dynamic framed-slotted ALOHA
EPC	Electronic product code
Es	Empty slot
FDMA	Frequency division multiple access
IIoT	Industrial internet of things
MAC	Medium access control
RFID	Radio-frequency identification
RN16	16-bit random number
SDMA	Space division multiple access
Ss	Successful slot
TDMA	Time division multiple access
UHF	Ultra high frequency
UII	Unique item identifier
